# Does Health Crises Effect Tourism: Role of Financial Inclusion for Green Financial Development

**DOI:** 10.3389/fpubh.2022.896894

**Published:** 2022-06-03

**Authors:** Qun Gao, Yun Liu, Bakhtawer Ayub, Mumtaz Hussain

**Affiliations:** ^1^Faculty of Economics and Management, East China Normal University, Shanghai, China; ^2^School of Finance, Shanghai Lixin University of Accounting and Finance, Shanghai, China; ^3^School of Mathematics, Institute of Southern Punjab, Multan, Pakistan; ^4^Center for Economic Research, Shandong University, Jinan, China

**Keywords:** health crisis, terrorism, financial inclusion, per capita income, GMM

## Abstract

Tourism is impacted by all types of crises, no matter how big or small. Even though many studies have examined tourism crises, most focus on the number of tourists arriving and departing. As a result of this lack of information, The adaptive differences in tourist behavior caused by various crises are not well understood. When it comes to inbound tourism, the financial and health-related crisis can significantly impact the tourist profile of the country and its visitors' spending habits. The findings show that the health crisis has a significant positive impact on tourism. Moreover, COVID_deaths and COVID_confirm_cases decrease the international tourism in developed and developing countries. According to the study's findings, tourists' sensitivity to crises varies between short- and long-haul markets. The evidence shows that financial inclusion has a significant positive impact on various aspects of tourism development in China. Hence, this article offers numerous policy and practical suggestions for sustainable tourism management.

## Introduction

It's no secret that the tourism industry is highly susceptible to and affected by various external and internal factors. Tourism demand and travel company performance can be adversely affected by an unpredicted disaster (e.g., natural, financial, and health). Pandemics and disease outbreaks may be the most significant because of their impact on social and economic change. Travel and tourism are more susceptible to disasters and crises than other industries (1). Strategies and measures for health communication (e.g., social distancing, travel and mobility bans, community lockdowns, stay-at-home campaigns, self- or mandatory quarantine, and crowding restrictions) have effectively halted international travel, tourism, and leisure (2–4). As a highly vulnerable industry to a wide range of environmental, political, and socio-economic risks, tourism is accustomed to and has become resilient in bouncing back (from various crises and outbreaks) from these risks (e.g., terrorism, earthquakes, Ebola, etc. SARS, Zika). However, the nature, the unprecedented circumstances, and the impacts of the COVID-19 show signs that this crisis is not only different, but it also has the potential to have profound and long-term structural and transformational changes to tourism as a socio-economic activity and industry, as well as to other industries. Indeed, the global and massive pandemic scale and the multidimensional and interconnected impacts that challenge current values and systems and lead to a worldwide recession and depression are the most distinguishing characteristics of this outbreak.

International tourist arrivals are expected to decline by 78 %, resulting in a loss of US$ 1.2 trillion in tourism-related export revenues and the elimination of 120 million direct tourism jobs, which is seven times the impact of September 11 and the largest decline in tourism employment ever recorded. Tourism and COVID-19 are at the epicenter of all international discussions and economies because they are among the most important global employers and major contributors to the GDP of several countries (5–7). There is a unanimous call to see and use the pandemic as a transformative opportunity in the burgeoning industry discussions and research about tourism and COVID-19. Likewise, the industry should not only recover but also reimagine and reform the next normal and economic order, and researchers should not solely use COVID-19 as a context in which to replicate existing knowledge for measuring and predicting tourism effects. Although such studies are critical for managing the pandemic, they do not advance understanding or guide the industry to a higher level of performance. Furthermore, because of the interconnected socio-cultural, economic, psychological, and political impacts of COVID-19 of this magnitude, it is expected that unexpected trajectories rather than historical trends will emerge, and the predictive power of 'old' explanatory models may be rendered ineffective. Furthermore, there is sufficient evidence to assert that both the tourism industry and research have matured significantly, resulting in insufficient knowledge about studying and conducting research efficiently. We still don't know how health crises can spur industry change, how companies can turn crisis disruption into transformative innovation, or how to conduct research that can help us think about and reset our expectations about what is normal for the future.

The tourism industry has yet to experience a major crisis that could serve as a catalyst for change. Crises have also been used to keep the status quo in place while limiting the scope for mass mobilization as a political tool. A crisis-driven transformation's nature and degree are dependent on how and whether these stakeholders are affected by and respond to crises, as well as how they recover and reflect after crises because change can be selective and optional for tourism stakeholders (such as tourists, operators, destination organizations) Consequently, tourism COVID-19 research needs to examine and understand more deeply the drivers, actions, and reactions of tourism stakeholders (behavioral, cognitive, emotional and even ideological) to COVID-19 impacts.

Next financial inclusion refers to all people and businesses should have access to financial services. In order to achieve the 17 United Nations Sustainable Development Goals (SDGs), the World Bank recognized the importance of this principle (8). Financial inclusion could theoretically have both a positive and a negative impact on the environment. Increasing financial inclusion could lead to a rise in energy-intensive appliances like refrigerators, televisions, and air conditioners, which would increase overall energy consumption and pollution, and carbon emissions (9). Improved financial inclusion can help farmers in poor communities instead of burning coal; consider switching to cleaner energy sources (such as microgrids of solar panels) (10). On the other hand, financial inclusion could boost innovation and technological progress in the tourism industry, reducing energy consumption and emissions (11). In addition, greater financial inclusion may make it easier for tourists to participate in high-pollution tourism, which could lead to fewer health crises and a reduction in CO_2_ emissions. For energy exploration and development to be successful, substantial funding may be required, which can be improved by ensuring more people access financial services (12).

Therefore, this study aims to investigate the impact of health crises on tourism. Another objective is to examine the role of financial inclusion in achieving the goal of environmentally friendly finance in tourism and crisis management literature by integrating an evolutionary approach. It is possible that financial inclusion could indirectly impact the tourism industry. As far as we know, no research has financial inclusion, and the tourism industry was examined. Regions differ significantly in emissions and financial inclusion, making regional differences in the impact of financial inclusion on the tourism industry and green financial development. However, very few studies have examined regional differences and mediation impact mechanisms. Thus, this study examines the impact of financial inclusion on tourism and green financial development and the emergence of health crises, using balanced panel data from 2000 to 2020. Researchers in health crisis management and tourism will benefit from this study's theoretical and practical contributions, showing how financial inclusion affects tourism and health. To put the findings of this research into practice, tourism professionals and policymakers should consider the type of crisis and the long-term effects on tourist spending and behavior.

The remainder of the article is structured as follows: Section Literature Review provides a literature review of current empirical research; Section Data and Methodology presents a case study. The technique and data are explained in part 3, and the empirical findings are discussed in depth in Section Results and Discussion. Finally, part 5 clarifies the study by outlining some policy suggestions based on the research's empirical findings.

## Literature Review

Tourism crisis studies in the past have focused primarily on the impact of a single crisis on tourist volume. Much less thought was given to how crisis types differed from one another and how tourists reacted during and after the crisis as patterns of change evolved. While researching the uncertainty avoidance approach, traveling through a crisis impacts tourists' behavior (13). An investigation by Aghaei et al. (14) based on the exploratory approach revealed that returning more experienced visitors are aware of the health and financial risks than first-time visitors. Because of this, it is critical to have a firm grasp on how tourists behave during various types of crises. As a result, tourism crisis research has a significant gap in understanding how the crisis affects tourist behavior and outcomes.

According to Lee and Brahmasrene (15), tourism in Greece, Italy, and Austria is hampered by health crises. There are “spillover effects” to health crises, and an outbreak in one country can affect tourism in neighboring countries. After the COVID outbreak, there was a decrease in the utilization of labor and capital. In Tunisia (16) study the impact of the COVID outbreak and financial inclusion on tourism. In Tunisia, they used both internal and external shocks to demonstrate that internal shocks have a greater impact on tourism activity. There was a significant drop in international tourist arrivals and spending in October and November of 2017 in Catalonia as a result of the political instability in the region. They conclude that Catalonia's tourist arrivals and spending decreased due to political events in the final three months of 2017. According to their findings, political flux has negative effects, even in the most stable countries. Research by Anup (17) examines how governance quality and political uncertainty affect tourist requirements. They used a gravity model to examine 131 countries as tourist origins and 34 countries as tourist destinations between 2005 and 2014. They discover that tourist traffic is greatly influenced by the quality of an area's institutions and the absence of political strife. Travel and tourism are harmed by institutional corruption, according to Woon Leong and Bee Lian (18), Karimi (19), and Wang and Wang (20) (tourist inflows, tourism competitiveness). It has been shown that corruption to combat bureaucracy can increase demand for tourism, as demonstrated by Gössling et al. (21) and Khanal (22).

Italy's domestic tourism flows are influenced by the environmental quality, according to Xiangyu et al. (23). Their study (24) found a two-way connection between environmental sustainability and tourism growth. According to these findings, changes in environmental sustainability policies and regulations can influence tourism growth. According to Jalil et al. (25), climate change has an impact on tourism, and countries that rely heavily on tourism are more vulnerable to the effects of climate change. According to Suhel and Bashir (26), poor air quality significantly impacts travel to five different European countries for pleasure or business (Austria, Switzerland, Cyprus, Great Britain, and Italy). Tourism and air quality appear to persuade each other, with poor air quality reducing tourism and poor air quality reducing tourism.

This paper examines how health is a major determinant of tourism. We investigate the significance of improved health care for a growing international travel market. There are a number of ways in which health can have an impact on the economy, as we explain below. Economic growth benefits from a healthy population have been documented in academic studies (27–29). According to Perles-Ribes et al. (30), human capital quality includes physical and mental wellbeing. In a sample of 100 countries, he shows that an increase in labor productivity correlates with an increase in life expectancy. In low-income countries (31), found changes in the overall survival of youths are linked to the rise in GDP growth. According to De Vita and Kyaw (32) health affects aggregate supply in a positive and statistically significant way. This is supported by Ohlan (33), that the average lifespan in Sub-Saharan Africa has a significant effect on economic growth. They've also found that health has a bigger impact on economic development than education. To better understand the impact of health investment on tourism and green financial development, Dogan and Aslan (34) conducted research. They found that green financial development is linked to both the growth rate in health and the level of health. Each country's medical status is measured using a standard and reliable method. Habibi et al. (35) investigate the relationship between tourism and health crises using life expectancy.

Health affects tourism in numerous ways. Note that infectious diseases (both pandemics and epidemics) have been thoroughly studied to see how they affect tourism (36, 37). They explore the challenges of organizing, replying, and restoring from a pandemic in a developing nation beyond the instant adverse effects of a pandemic on tourism in a small developing country. According to Narayan et al. (38), the pandemic effect of COVID-19 is compared to other epidemics and pandemics to see if the economy and tourism are affected. They demonstrate how tourism is sensitive to measures designed to limit its spread, such as mobility restrictions. The pandemic has had a devastating impact on the economy and tourism. Final thoughts: The COVID-19 pandemic should prompt a rethink of how tourism is grown globally, taking into account climate change and its dangers. A study by Blake (39) calculates the impact on Asian countries of two infectious diseases (Avian Flu and Severe Acute Respiratory Syndrome, SARS). They concluded that SARS had a more significant impact on Asian tourism than the Avian Flu has. Compared to the Avian Flu, SARS was more infectious. These results are logical. The 2019 covid-19 pandemic has decimated the tourism sector in every country, according to the UNWTO (40). Each country— Small Island asserts, then the least developed countries, and too many African states—has been devastated by the global financial crisis. The pandemic of covid-19 has slowed progress toward the UN's 17 long-term development goals (SDGs). The literature has extensively studied the impact of health crises on tourism.

### Global Recession, Epidemics and Tourist Trends

In and around destinations, shock and stress are felt during a crisis regardless of the event's type, probability, or magnitude. Studies have shown how the crisis affects the destination, awareness, feedback and adaptations to the crisis, and spending patterns (41–43). Tourism crisis research has primarily focused on monetary and health issues. The financial crisis has gotten more academic attention for the first time because it is linked to socioeconomic issues such as unemployment (44, 45). As a result, job insecurity and the resulting decrease in available spending power heavily influence people's travel decisions. The tourism industry has been the focus of previous studies on SARS. SARS or the avian flu may threaten people's health, safety, and wellbeing, but it also poses difficulties for the travel industry, particularly inbound travelers. In this sense, it is comparable to a financial meltdown.

Tourists' travel plans and activities are influenced by a crisis in numerous ways, from before, during, and after the crisis. According to Wang and Wang (20), tourists prefer to stay in low-cost hotels and rooms during economic uncertainty. When it comes to tourist demand in a recession, income and personal economic situation have little or no effect, according to Robaina-Alves et al. (46). They also noted that the European financial crisis did not deter Europeans from taking vacations. Middle-aged and experienced travelers, according to Zhang and Zhang (47), are less sensitive to a crisis than younger and less experienced travelers. No current evidence explains why tourists react differently when confronted with a crisis. However, the impact of a crisis on tourism varies depending on the type of visitor and their goals. According to Ming Cheng et al. (48), health crises (such as food security) are more important to first-time visitors than repeat visitors' financial risks, indicating a motivational divide between the two groups. According to Wieczorek-Kosmala (49), women are more concerned about their health and nutrition than men are. It has become more common for people to spend their vacations at home rather than going out to eat more often, buy more private labels, and entertain at home more often. According to Luo and Deng and Ramukumba (42, 44). From a broader perspective, the current state of the economy restricts tourists' ability to travel further.

These previous tourism crisis studies, on the other hand, neglected to take into account how crises evolve. In most studies, only the time of the crisis is examined, ignoring changes before, after, and during the disaster. Instead, they concentrate with respect to just one instance and how it affects the number of tourists. Crisis type, tourist profile, purpose, and pattern all play a role in how it affects tourist behavior. So, more research is needed to look at the impact of health crises and changes from a tourist's point of view.

### Crisis in Tourism

In contrast to disaster, which is the result of action outside of the body that may have an immediate effect, “crisis” refers to incidents that are more closely tied to the company's environment (41). The tourism crisis is deeply rooted in economics, sociology, the environment, and politics. Both crises and disasters significantly impact the tourism industry regarding reduced tourist flow, travel plans by tourists, unemployment, and investment by businesses. Luo and Deng (42) also points out that a crisis occurs as a result of a failure to respond to a disaster. There has been a lot of attention paid to disasters and crises in tourism studies, but few studies have focused on the economic crisis. For example, a theoretical model that examines the connections between health crises (41), tourism competitiveness, and green financial development was developed recently (43, 50). It has been shown that tourism competitiveness is influenced by the health crisis when an economic crisis occurs in Spain (51, 52). No one knows much about the impact of disaster and crisis on tourism, particularly the evolutionary differences in tourist behavior caused by different crises, despite findings published by Korstanje and Tarlow (53).

Because of the uncertainty, disruption, and risk accompanying health crises (54), health crises alter behavior and normal tourism trends. With the recent Arab uprisings, tourism in the Middle East and North Africa was put at risk and negatively impacted tourism development. Arab uprising effects vary even among Mediterranean countries, as revealed by Atan and Arslanturk (55) in their study Puri et al. (56). There has been a significant reduction in tourism to the Middle East and North Africa as a result of the ongoing Syrian conflict (57). The crisis has a greater impact on inbound tourism than outbound tourism (58). For example, incoming tourism to the Middle East is still at risk due to the high probability of terrorist attacks. A crisis can significantly impact declining tourist numbers, the use of facilities at tourist destinations, and an imbalance between supply and demand.

### Financial Inclusion, Green Financial Development, and Tourism

For example, according to a study conducted in Turkey by Chen et al. (59), the growth of financial markets has a significant impact on tourism development and expansion. According to their empirical analysis, foreign direct investment and trade play a mediating role in the underlying connection between the tourism industry and economic growth, spanning 1960 to 2015. Also, in Malaysia, Lei et al. (60) found that tourism demand increased as a result of improvements in financial development while taking into account the country's structural data breaks. Çaglayan et al. (11) report mixed effects of China's economic development on the country's tourism growth in a study looking at how it affects both the sector's positive and negative outcomes. They found that financial development causes tourism growth. In contrast, Abdullah et al. (61) green tourism is directly linked to financial development and vice versa, based on data collected from 2000 to 2016.

A lack of research has examined how regional tourism demand has been affected by financial development, which includes several countries beyond the country level. For example, according to Feng et al. and Lv et al. (62, 95), the long-term demand for tourism to Japan, South Korea, and Singapore will be increased by green economic literacy, especially in the short term. Nabeeh et al. (63) Research has found a causal connection only in high-income countries in the United States but not Europe or Asia. The link is established between international tourism to financial services.

Finally, Punzi (64) observed the link between tourism specialization and economic expansion in one of the few reported studies that performed a panel analysis for a larger number of nations (129) over the timeframe 1995–2011. Their research examines whether this relationship is linked to a host country's financial development. As it turns out, green financial development lessens the link between tourism dependence and public health crises. Still, the benefits are diminishing in those countries with high levels of financial development, and a heavy reliance on tourism tends to suffer economic setbacks.

To the best of our knowledge, no studies have empirically examined the impact of financial inclusion on tourism development despite the body of literature on the topic expanding rapidly. As we mentioned in the introduction (65, 66), are the sole notable exception. Due to its similarity to our work, this paper deserves further discussion. At least two distinct dimensions must be considered when assessing financial sector development: financial sector depth and financial inclusion. A plethora of literature has already established this point. The former typically refers to a country's overall development in banking, equity, and bond markets. In contrast, the latter refers to the provision of access to and use of financial services. The failure of a Debrah et al. and Wang et al. (67, 68) to distinguish between these two dimensions implies that their results cannot differentiate between the effects of financial inclusion and the impact of the depth and efficiency of the green financial market.

This ambiguity necessitates establishing a link between economic growth and tourism demand can be seen. Academic studies have discovered a substantial change in macroeconomic and development outcomes between financial inclusion and depth or efficiency. This is important in light of these findings (69). Since there is still a significant gap in the literature on whether financial inclusion's unique effects influence tourism demand, it is important to address this issue in tourism development.

## Data and Methodology

### Empirical Model

For this purpose, the following instrumental variable econometric model is used to analyze the health crisis's effect on tourism. The system GMM model is used to estimate our model. Unobserved nation impact, the endogeneity of control variables, the correlation between independent variables and past or possible current error realizations, and heteroskedasticity and autocorrelation within individuals are addressed by this assessor (70).

Estimates for international tourism receipts are made using the following model specification:


(1) $$y_{it}=\lambda y_{it-1}+\beta x_{it}+\gamma z_{it}+\eta_{i}+\varepsilon_{it}$$

(2) $$\;u_{it}=\eta_{i}+\varepsilon_{it}$$

$$\eta_{i}∼IID(0,\sigma_{\eta }^{2}),\varepsilon_{it}∼IID(0,\sigma_{\varepsilon }^{2})\text{ and }E[\eta_{i}\varepsilon_{it}]=0$$

The dependent variable yit is a measure of the revenue received from tourists from outside the country; the lagged variable y (it-1) represents the lagged dependent variable; the vector zit contains control variables; the vector xit contains explanatory variables; and the error term, u it, includes the variables I and _it; random disturbance term _it; unobserved country-specific effects or fixed effects where is correlated with y (it-1). A first difference transformation of Equation (1) by Tolkach (71) eliminated the unobserved effects:


(3) $$\Delta y_{it}=\lambda \Delta y_{it-1}+\beta \Delta x_{it}+\gamma \Delta z_{it}+\Delta \varepsilon_{it}$$

Taking the first Difference in Equation 2 involves:


$$\begin{array}{l} \Delta u_{it}=\Delta \eta_{it}+\Delta \varepsilon_{it}\Rightarrow u_{it}-u_{it-1=}(\eta_{i}-\eta_{i})\\ \;+(\varepsilon_{it}-\varepsilon_{it-1})\Rightarrow \Delta u_{it}=\varepsilon_{it}-\varepsilon_{it-1} \end{array}$$

The first operator of Difference is. There is a new bias that the differenced error term can be correlated with the lagged dependent variable after the first differencing. The system GMM estimator was proposed by Danish & Wang (72) and Phoochinda (73). In this system, A lagged first difference is used to instrument the explanatory variables and a level of one in between each of those variables, using two equations, one first-differenced and one in groups. For the system estimator to work, variables in levels and differences are both instrumented with the lags of their levels and vice versa. Level moment conditions in System GMM supplement the first differenced moment conditions of Difference GMM, as noted by Arzhang and Hamidi (74), for improved estimation efficiency. The System GMM estimation has two diagnostic tests to ensure its accuracy. There are two ways to test the validity of the instrument set: the Sargan test for over-identification and the Arellano–Bond (AR2) autocorrelation, both of which were proposed by Korstanje and Tarlow (53). Stata 16's System GMM estimation can be performed with the xtabond2 command Santana-Talavera and Fernandez-Betancort (75).

### Data, Variable and Descriptive Statistics

The empirical analysis is based on a cross-sectional dataset containing data from 97 developed and developing countries. The availability of data dictates the countries that are considered. This study incorporates data from a variety of sources, including the World Development Indicators (WDI), the World Travel and Tourism Council (WTTC) (WTTC, 2020), and the F.M. Global and Associates (F.M. Global and Associates, 2019) (2020).

Tourism: A country's tourism industry can be gauged using five different metrics: the amount of money spent on leisure travel, the amount spent on business travel, the amount of money spent on travel and tourism that goes directly into GDP, and the number of tourists who cross the country's borders (in millions). For the purpose of capturing the tourism variable, we use the international tourism receipts in US dollars expressed in current prices. based on the World Development Indicators published by the World Bank (WDI). Health crises measures: In this study, the outbreak of COVID-19 and previous non-COVID health crises are used to define health crises in a tourist destination country. WDI indicators are also used. This is in addition to OECD (2011) using COVID-based and non-COVID-based diseases to define a country's health crisis. Green financial development: Green financial development is also linked with better health conditions in any country. Green financial development means the inflow of investment to reduce the CO_2_ emission in the environment. Financial wellbeing is necessary for businesses and health institutions to combat the COVID and non-COVID health crises for good health conditions. The number of COVID-19 deaths and cases that have been confirmed reveals the severity of the health crisis. Financial inclusion: It is possible to construct the financial inclusion (FI) index using information about the depth, accessibility, and efficiency of financial institutions and financial markets. In addition, the study takes into consideration three other important determinants of tourism development, including GDP; trade openness, which is measured using information on total trade in goods and services, and trading partners' diversity (exports and imports).

## Results and Discussion

This research investigates how health crises affect the tourism industry and the role of financial inclusion in green financial development for developing and developed countries. Throughout the study period, the descriptive statistics for the sample variables in developing and developed countries can be found in Table 1. The tourism has a standard deviation of 28.693 in sample countries, while the COVID-deaths and COVID-confirm cases variable has a standard deviation of 0.1182 and 0.8147 in these countries. The mean of the trade variable is 74.2827. According to the descriptive statistics, the standard deviation of the trade variable is 35.7528. The mean for trade is also the highest, coming in at 100.9534 points.

**Table 1:** Descriptive statistics.

**Variables**	**Mean**	**Std. Dev**.	**Min**.	**Max**.	**Mean**	**Std. Dev**.	**Min**.	**Max**.
Tourism	21.4188	1.5056	17.5767	24.6834	22.5568	1.4628	17.767	26.2414
COVID-deaths	4.2475	0.1182	3.8344	4.4117	4.35	0.0552	4.1751	4.4305
COVID-confirm	3.2263	0.8147	1.2237	5.2192	1.7452	0.539	0.6931	3.7565
Non-COVID	0.6405	1.1644	0.01	6.92	1793	0.0169	0	0.12
CO2	0.8264	0.6318	1.1086	1.9198	1.6702	0.4071	0.0295	2.6369
FI	2.2289	0.7743	0.9162	3.6216	1.0337	0.3035	0.9162	3.1696
FD	17.0903	21.9899	0.18	85	3.0374	5.2452	0	29
GDP	8.3537	1.0081	6.2578	11.0548	10.1075	0.8809	7.2473	11.6259
TRADE	74.2827	35.7528	20.72	220.41	100.953	59.9264	19.8	416.39

### Discussion of Empirical Results

The System GMM estimation results of developing and developed countries are presented in Tables 2, 3. Each panel of developing and developed countries is subjected to six regression analyses, separately using the health feature variables. We also examine the impact of international tourism revenue on the rising quality of health in developed countries. Lagged variable for international tourism is positive and significant. International tourism have an important impact on achieving more international tourism. The findings of the study are in line with Su (76). Strongly positive and significant are the results of the variable. This implies that international tourism could rise as a consequence of a rising average lifespan or that an increase in life expectancy is correlated with an increase in revenue from international tourism. For developing countries, the findings shows that an increase in COVID_confirm_cases of 1 % will result in an decrease of 0.427 % in international tourism, while for developed countries, 0.406 % decrease in international tourist receipts. According to Pulicherla et al. (77), for medium and low-income countries, a country's average lifespan is an essential element of its safety.

**Table 2:** Effects of Health crises on tourism in developed countries.

**Dependent variable** **=** **Tourism**			
**Variables**	**(1)**	**(2)**	**(3)**
Tourism_Lag1	1.010	0.952	0.809
	(0.002)	(0.004)	(0.001)
COVI_deaths	−0.271		
	(0.003)		
COVI_confirm_cases		−0.427***	
		(0.012)	
Non_COVID			0.002***
			(0.005)
FI	0.111***	0.099***	0.086***
	(0.000)	(0.000)	(0.000)
FD	0.026***	0.039**	0.061**
	(0.046)	(0.037)	(0.049)
GDP	0.011***	0.013***	0.006***
	(0.000)	(0.000)	(0.047)
Trade	0.034***	0.038***	0.027***
	(0.000)	(0.004)	(0.597)
Arellano–Bond Test AR(1)	[0.002]	[0.013]	[0.000]
Arellano–Bond Test AR(2)	[0.118]	[0.289]	[0.078]
DiffinHansen test	[0.952]	[0.682]	[0.611]
Hansen J test	[0.772]	[0.635]	[0.400]

***1% significance level.

**Table 3:** Effects of Health crises on tourism in developing countries.

**Dependent variable** **=** **Tourism**			
**Variables**	**(4)**	**(5)**	**(6)**
Tourism_Lag1	0.959***	0.905***	0.768***
	(0.002)	(0.004)	(0.001)
COVI_Ddeaths	−0.257***		
	(0.003)		
COVI_Dconfirm cases		−0.406***	
		(0.011)	
Non_COVID			0.002***
			(0.015)
FI	0.105***	0.094***	0.081***
	(0.000)	(0.000)	(0.000)
FD	0.025*	0.037**	0.058**
	(0.066)	(0.033)	(0.047)
GDP	0.310***	0.312***	0.306***
	(0.000)	(0.000)	(0.005)
Trade	0.018***	0.013***	0.006***
	(0.000)	(0.003)	(0.002)
Arellano–Bond Test AR(1)	[0.002]	[0.013]	[0.000]
Arellano–Bond Test AR(2)	[0.112]	[0.275]	[0.074]
DiffinHansen test	[0.904]	[0.648]	[0.580]
Hansen J test	[0.734]	[0.603]	[0.380]

***1% significance level.

This study found that a 1% decrease in COVID_deaths results in a 0.271% decrease in international tourism, while, the association among COVID_deaths and tourism is negative and statistically significant, a 1% increase in COVID_deaths, results in a 0.257% decrease in tourism in the developing countries. It shows the average lifespan is a significant and strong negative. International tourism revenue could be advanced in countries with a low mortality rate if they focus their hard work on reducing the death rate.

International tourism receipts are positively affected by the non-COVID variable. The risk of maternal death negatively impacts global tourism receipts over the course of one's life. Development countries' international tourism revenue will drop by 0.427%. Domestic government health expenditures have tourism revenues in emerging regions have a positive but not statistically significant influence. The government's spending on health care in developed countries has a considerable impact on the amount of money from international tourism. As a result, developed countries' high public health expenditures and high tourism revenues go hand in hand. Cui et al. (78) support this conclusion. For developing countries, the emission of CO_2_ is a highly negative and significant variable. In other words, as the prevalence of malnutrition rises, international tourism revenue may fall. To attract more tourists and raise their income, developing countries must reduce the number of people living in poverty.

As a result, the health quality variable in developing countries is marginally negative but significant. In other words, a high death toll from tuberculosis hurts the country's ability to attract tourists from around the world. If the death toll from tuberculosis rises by 1%, international tourism revenues will fall by 0.0295%. The death toll from tuberculosis has a downbeat blow on international tourism revenue, but this is not statistically significant when comparing developed and developing nations. Negative and significant health quality components are the most important. This suggests that a decrease in international tourism receipts is linked to the decline in the quality of health in both developed and developing countries. Moreover, GDP, financial inclusion and financial development in most developed and developing countries have positive and significant on tourism. According to numerous studies, real GDP per capita has a positive impact on international tourism receipts (79).

Revenues from travel and tourism abroad in developing countries are positively impacted by trade openness, although in a small but meaningful way. International tourism receipts are expected to rise as a result of more significant free trade. Trade openness has a small but positive impact on international tourism receipts in developed countries, but it is still statistically significant. However, this is consistent with the findings of Feng et al., Ohajionu et al., and Rowan and Galanakis (62, 80, 81).

The relationship between worldwide trade and tourism revenues is weak but not important, as has been constantly observed by many previous studies. Lv et al. (96) and Sarangi (82) have found similar results. As a result of the perceived effect of exchange rates on tourism industry revenues, we expect to increase international tourism revenue. The supply chain of the “tourist destination” region is enhanced by a reduction in trade terms. Wage growth has a small but noticeable effect on foreign tourism revenue in developed and emerging economies. It's safe to say that the financial risk factor is both present and significant. As a result, developing and developed countries will see an increase in their revenue from international tourism when financial risk is lower. Because of this, reduced financial exposure should be a priority for all countries, developing and developed alike. Incorporating the 2008–2009 global economic crises as a model variable resulted in interesting empirical findings. The financial crisis dummy variable negatively impacts tourism receipts. So, it's safe to say that the recent global financial crisis has had an effect on travel to emerging markets. Research by Cui et al. and Wang and Zhi (78, 83) supports this.

### Robustness Test

Additionally, as a robustness check, we also examine the effect of additional control variables on our results. Our re-estimation of the main model takes into account the proportion of the population over 65, the log of population density, and the log of the urban population as a % age of the total population. The total number of confirmed deaths and cases was used instead of the fatality rate as an additional explanatory variable. Table 4 summarizes the findings from this experiment. The key explanatory variables show no significant shifts in their signs or significance. With the proportion of people over 65 and confirmed deaths, tourism has a positive and significant correlation (84).

**Table 4:** Robustness test.

**Variables**	**Tourism spending**	**Tourist arrivals**	**Tourism receipts**	**Tourism contribution to GDP**
Tourism_Lag1	0.614***	0.689***	0.639***	0.613***
	(0.279)	(0.289)	(0.296)	(0.292)
COVI_Ddeaths	−0.681***	−0.420***	−0.632***	−0.347***
	(−0.920)	(−0.930)	(−0.798)	(−0.527)
COVI_Dconfirm cases	−0.067	−0.117	−0.621	−0.239
	(−0.089)	(−0.089)	(−0.770)	(−0.526)
Non_COVID	0.713***	0.687***	0.606***	0.784***
	(0.058)	(0.058)	(0.059)	(0.058)
FI	0.026**	0.008***	0.018***	0.006***
	(0.063)	(0.063)	(0.067)	(0.063)
FD	0.041*	0.042**	0.037**	0.047**
	(0.025)	(0.024)	(0.025)	(0.024)
GDP	0.788***	0.777***	0.797***	0.747***
	(0.046)	(0.046)	(0.045)	(0.044)
Trade	0.043**	0.044**	0.039**	0.047***
	(0.110)	(0.110)	(0.112)	(0.110)
Constant	0.799***	0.799***	0.362***	0.877***
	(1.381)	(1.370)	(1.392)	(1.289)

*Errors in parentheses of a robust standard*.

^***^p < 0.01,

^**^p < 0.05,

^*^p < 0.1.

It's also possible to make the case that a relative rather than an absolute indicator of the health of the tourism industry is preferable to look at. We double-checked using the ratio of tourists visiting a country *i* to the total number of tourists visiting all of our sample countries or the ratio of visitors to the country *i* to total tourists visiting by region for all the countries included in the sample robustness of our estimates. Logically, a country's tourism sector is more significant if the value of these ratios is > 0. Table 4 displays the exercise's outcomes. When it comes to signs and magnitudes, consistent with initial estimates, the variables of interest's limits are generally estimated in the same way.

### Changes in Tourist Behavior Patterns

Among the immediate concerns is the effect of COVID-19 on the geographic distance of travel. More than 70% of those polled said they wanted to travel more within the United States. Only a sliver of those surveyed said they wanted to travel more internationally. According to the research, 23.1% of people are increasingly interested in traveling alone, with women showing a slightly higher interest (25.0%) than men (20.8%). At the same time, most respondents (68.7 %) will use tourist intermediaries as before. Only 19.8 % and 11.5 % plan to decrease or increase their use in planning their trip.

Of those polled, 47% stated that they would continue to contract the same type of insurance before COVID-19, but 34.4 % stated their insurance needs had increased rather than decreased, while just 17% stated the opposite. It's also worth noting that women (38.0 %) have a higher proclivity than men (35.0 %) to obtain additional insurance (31.6 % ).

While most respondents (71.4 %) expect their vacations to be the same length as last year, a sizable minority (27.0 %) intend to cut back on their time away. As a result, 38.3% of respondents plan to alter their usual travel schedules. According to the gender of the respondents, there were significant differences in the results. 42.5 % of women would change their dates, compared to 33.0 % of men, in this situation. According to these findings, those willing to change their travel dates are remarkably similar in age and sex (2 = 11.904; p = 0.018) (see Table 2).

### Public Social Welfare

Pandemics have a negative impact on tourist arrivals in 97 countries. Only low-income countries are affected by this effect. Traveler arrivals are expected to fall by 2.1% for every 10% increase in the discussion about the pandemics index. When the pandemic is active, the index value rises dramatically, and countries that experience a drop in visitor numbers suffer economically. In addition, the impact of pandemics on fast and slow-growing tourism destinations will be different.

Because the world has never seen a pandemic of this magnitude, it is difficult to predict how COVID-19 will affect it. The governments are implementing travel restrictions and border closures. As a result, our findings from previous pandemics will only have limited utility in predicting the potential effects of COVID-19 on tourism. Using data from a single country or a group of countries on a quarterly/yearly basis, Tourist arrivals can be studied in future studies using the autoregressive distributed lag model to examine the short and long-term effects of pandemic outbreaks (85).

A statistically significant and positive effect on tourism arrivals is found, indicating that inbound tourism is persistent at a high level. In columns 7–9, the estimates are based on various income levels. The negative impact of pandemics on tourist arrivals disappears for emerging and advanced economies, and it remains only for low-income economies. There may be fewer tourists visiting low-income countries because of the lack of openness and inadequate health care facilities. The previous pandemics in recent history were confined to a small geographic area and caused far fewer deaths and illnesses than the coronavirus. As a result, the health systems of advanced and emerging economies could handle the pandemics. Travel bans and curfew restrictions were not widely implemented. On the other hand, Viruses have become more global in scope as a result of increased globalization. COVID-19-related deaths and infections are rising, and countries worldwide are enacting travel restrictions, quarantines, and curfews to stem the tide. A flattening of the COVID-19 curve is needed because the health care systems in nearly every country are on their way to failure. Because of this, Kleih et al. (86) argue that the impact of COVID-19 will be unique, making it difficult to extrapolate from previous experiences.

Tourist arrivals are positively correlated with GDP per capita in all estimations. Tourist arrivals increase as GDP per capita rises. Tourist arrivals increase when the exchange rate is favorable. The findings are consistent with previous research (85). Trade openness positively impacts tourist arrivals, implying that countries that integrate with the rest of the world are more likely to attract tourists. It is shown in Table 3 that pandemics have a long-term impact on tourism. For all estimates, there is no evidence of a lag effect. There were no long-term effects of the previous pandemics on tourist arrivals because they were short-lived.

## Conclusion and Policy Recommendations

The spread of the COVID-19 pandemic has necessitated implementing physical distance measures and mobility restrictions, including international travel bans, in many countries. These restrictions have slowed economic activity around the world and affected millions. Economic stimulus packages were implemented in both developing and developed countries in order to counteract the negative effects of the economic impact of the pandemic. Economies that rely heavily on physical interaction and flexibility are the worst hit. The COVID-19 pandemic has had a devastating effect on the tourism industry. Pandemic effects on economies worldwide would be devastating, most notably in tourist-dependent nations to create jobs, generate revenue, earn foreign trade, and stimulate financial expansion (87–89).

Because the COVID-19 pandemic has ravaged the tourism industry, we believe that economic stimulus packages in countries that rely heavily on tourism are more likely to be large than those that don't. However, Economic stimulus responses will be moderated by the degree of country resilience, better prepared for disasters than less resilient countries than low-resilient ones to respond to and recover from shocks, such as the COVID-19 eruption. Because COVID-19 has a more significant impact on tourism businesses in high-resiliency countries, governments will need to provide less financial assistance to them.

As a result of the COVID-19 pandemic, countries with a higher economic resilience introduced smaller stimulus packages. As a result of these findings, countries that are more resilient overall, heavily reliant on smaller economic stimuli, and less reliant on tourism packages are more likely to be implemented in tourist countries than countries with a lower resilience index. As a result, countries with high economic resilience that are also heavily dependent on the tourism sector have lower economic stimulus packages than those not highly reliant on the tourism sector. Tourism-dependent economies introduced smaller stimulus packages than those without such high-risk qualities, further reducing their reliance on the tourism sector.

High-resilience countries are better prepared to deal with shocks like the COVID-19 pandemic. Tourism businesses in countries with a high level of flexibility were enhanced to deal with the COVID-19 pandemic and thus required less government support than those with lower levels of resilience. Compared to the tourism industry in countries with a lower level of stability, those in highly resilient nations are less reliant on government incentive packages because Worthwhile human wealth and a more strong labor market make them better able to access financial markets and banks (90).

According to the study, tourism businesses can better respond and recover from the COVID-19 pandemic, for example, if the country's buoyancy is strengthened. Improved health crisis management and a conducive political, corporate, and economic environment can help countries become more resilient to natural disasters and improve their tourism industries' ability to withstand them.

Lastly, when dealing with the COVID-19 pandemic, researchers are debating whether or not a radical shift toward sustainability is necessary. This study's findings add to that discussion (91–93). According to Işik et al. (94), resilience is essential for long-term viability. We found that country-level stability can help governments protect tourism businesses from the COVID-19 pandemic at a lower cost, which is reliable with this notion. Due to smaller economic stimulus packages being introduced, tourism companies with high adaptability may be better able to withstand disruptive events, such as the COVID-19 pandemic. Therefore, High-resilience tourist destinations' tourism industries may be more long-term than those in countries with a lower level of resilience; therefore, further research should focus on the supporting policy responses that support tourism's sustainable transformation in the role of country-level strength.

## Data Availability Statement

Publicly available datasets were analyzed in this study. This data can be found here: WDI.

## Author Contributions

All authors listed have made a substantial, direct, and intellectual contribution to the work and approved it for publication.

## Conflict of Interest

The authors declare that the research was conducted in the absence of any commercial or financial relationships that could be construed as a potential conflict of interest.

## Publisher's Note

All claims expressed in this article are solely those of the authors and do not necessarily represent those of their affiliated organizations, or those of the publisher, the editors and the reviewers. Any product that may be evaluated in this article, or claim that may be made by its manufacturer, is not guaranteed or endorsed by the publisher.
